# Supplementary material for: Circadian Rhythm and Psychiatric Features in Wolfram Syndrome: Toward Chrono Diagnosis and Chronotherapy
Source: Diagnostics (Basel). 2025 Sep 15;15(18):2338. doi: 10.3390/diagnostics15182338 (PMC12468670; doi:10.3390/diagnostics15182338)
Supplement: Supplementary file 1 [file diagnostics-15-02338-s001.zip › diagnostics-3791950-supplementary.pdf]

diagnostics

MDPI

Supplementary Meterials

Table S1. Documents retrieved from each database and number of studies meeting inclusion and exclusion critria. Source: Authors' screening log.

Database	**Documents Retrieved**	**Meeting Inclusion and Exclusion Criteria**
Scopus	843	8
Web of Science	672	6
Dialnet	116	5
PsycInfo	36	2
PsicoDoc	29	1
ScienceDirect	125	5
Total	1821	27

Table S2. References analyzed in the scoping review, including study titles, authors, and publication years. No. (Ref): Reference number as cited in the main bibliography. Source: Author's elaboration.

No. (ref)	Title	Authors	Year
5	Selective cognitive and psychiatric manifestations in Wolfram Syndrome	Bischoff AN, Reiersen AM, Buttlaire A, Al-Lozi A, Doty T, Marshall BA, Hershey T	2015
6	Psychiatric findings in Wolfram syndrome homozygotes	Swift RG, Sadler DB, Swift M	2019
7	Psychiatric diagnoses and treatment in Wolfram Syndrome [abstract].	Reiersen AM, Narayanan A, Sinkre RA, Hershey T.	2019
8	Psychiatric disorders and mutations at the Wolfram syndrome locus.	Swift M, Gorman Swift R.	2000
13	Wolfram syndrome: Portuguese research	Ferreras C, Gorito V, Pedro J, et al.	2021
15	Correlates of depression and anxiety in patients with different rare diseases	Uhlenbusch, N., Löwe, B., & Depping, M. K.	2018
16	Depression and anxiety in patients with different rare chronic diseases: a cross-sectional study.	Uhlenbusch N, Löwe B, Härter M, Schramm C, Weiler-Normann C, Depping MK, et al.	2019
17	Síndrome de Wolfram	Silva P, Cedres L, Vomero A, Tapie A, Rodríguez S, Raggio V, et al.	2019
18	Síndrome de Wolfram: reporte de casos	Welschen D, Peralta Alejandro MC, Arroyo Yllanes ME, Fonte-Vázquez A.	2015
19	Síndrome de Wolfram - Relato de caso	Ferreira VFS, Caixeta LF, Caixeta M, Aversi-Ferreira TA.	2021
20	Organic mood syndrome in two siblings with Wolfram syndrome	Nanko S, Yokoyama H, Hoshino Y, Kumashiro H, Mikuni M	1992
21	Psychiatric disorders in 36 families with Wolfram syndrome	Swift RG, Perkins DO, Chase CL, Rush AJ, Amador X	1991
19	Bipolar Disorder Type 1 in a 17-Year-Old Girl with Wolfram Syndrome	Xavier J, Bourvis N, Tanet A, Perisse D.	2016
23	Mania in wolfram's disease: From bedside to bench	Chatterjee SS, Mitra S, Pal SK	2017

24	Wolfram Syndrome and Suicide: Evidence for a Role of WFS1 in Suicidal and Impulsive Behavior	Sequeira A, Kim C, Seguin M, Lesage A, Chawky N, Desautels A, et al.	2003
25	Psychiatric symptoms in a patient with Wolfram syndrome caused by a combination of thalamic deficit and endocrinological pathologies	Nickl-Jockschat T, Kunert HJ, Herpertz-Dahlmann B, Grözinger M.	2008
26	Wolframin mutations and hospitalization for psychiatric illness	Swift M, Gorman Swift R	2005
27	Psychiatric disorders in Wolfram syndrome heterozygotes	Owen MJ	1998
28	An atypical case of late-onset Wolfram syndrome 1 without diabetes insipidus	Rigoli L, Caruso V, Aloi C, Salina A, Maghnie M, d'Annunzio G, et al.	2022
29	Multidimensional analysis and therapeutic development using patient iPSC–derived disease models of Wolfram syndrome	Kitamura, R. A., Maxwell, K. G., Ye, W., et al.	2022
30	Wolfram Syndrome Type 2: A Systematic Review of a Not Easily Identifiable Clinical Spectrum	Rosanio FM, Di Candia F, Occhiati L, Fedi L, Malvone FP, Foschini DF, et al.	2022
31	A mutant wfs1 zebrafish model of Wolfram syndrome manifesting visual dysfunction and developmental delay	Cairns G, Burté F, Price R, O'Connor E, Toms M, Mishra R, et al**.**	2021
32	Wolfram syndrome 1b mutation suppresses Mauthner-cell axon regeneration via ER stress signal pathway	Wang Z, Wang X, Shi L, Cai Y, Hu B.	2022
33	Novel mutations in the WFS1 gene are associated with Wolfram syndrome and systemic inf lammation	Panfili, E., Mondanelli, G., Orabona, C., et al.	2021

34	Delineating Wolfram-like syndrome: A systematic review and discussion of the WFS1-associated disease spectrum	de Muijnck, C., Brink, J. B. T., Bergen, A. A., et al. (2023).ç de Muijnck C, Brink JBT, Bergen AA, Boon CJF, van Genderen MM, van den Born LI, et al.	2023
35	Clinical Peculiarities in a Cohort of Patients with Wolfram Syndrome 1	Salzano G, Rigoli L, Valenzise M	2022
36	Psychiatric Diagnoses and Medications in Wolfram Syndrome	Reiersen AM, Noel JS, Doty T, Sinkre RA, Narayanan A, Hershey T.	2022
